# Shikimic acid protects against doxorubicin-induced cardiotoxicity in rats

**DOI:** 10.1038/s41598-025-90549-4

**Published:** 2025-03-08

**Authors:** Maha Abdullah Alwaili, Amal S. Abu-Almakarem, Karim Samy El-Said, Thamir M. Eid, Maysa A. Mobasher, Ashwaq Hassan Alsabban, Najla Ali Alburae, Abeer A. Banjabi, Mohamed Mostafa Soliman

**Affiliations:** 1 Department of Biology, College of Science, Princess Nourah bint Abdulrahman University, 11671 Riyadh, Saudi Arabia; 2 Department of Basic Medical Sciences, Faculty of Applied Medical Sciences, Al-Baha University, Al-Baha, Saudi Arabia; 3 Biochemistry Division, Chemistry Department, Faculty of Science, Tanta University, Tanta, Egypt; 4 Department of Biochemistry, Faculty of Science, King Abdulaziz University, 21589 Jeddah, Saudi Arabia; 5 Department of Pathology, Biochemistry Division, College of Medicine, Jouf University, 72388 Sakaka, Saudi Arabia; 6 Department of Biological Science, Faculty of Sciences, King Abdulaziz University, Jeddah, Saudi Arabia; 7 Unit of Neurological Disorders, Department of Genetic Medicine, Faculty of Medicine, Princess Al-Jawhara Center of Excellence in Research of Hereditary Disorders (PACER.HD), King Abdulaziz University, Jeddah, Saudi Arabia; 8 Department of Biology, College of Science, Jazan University, Kingdom of Saudi Arabia, P.O. Box. 114, 45142 Jazan, Saudi Arabia; 9 Department of Zoology, Faculty of Science, Ain Shams University, PO Box 11566, Cairo, Egypt; 10 Biochemistry Department, Faculty of Science, Tanta University, Tanta, Egypt

**Keywords:** Shikimic acid, Antioxidants, Doxorubicin, Docking cardiotoxicity, Biochemistry, Chemical biology, Molecular biology

## Abstract

Doxorubicin (DOX) is used to treat a variety of malignancies; however, its cardiotoxicity limits its effectiveness. Shikimic acid (SA) showed several promising biomedical applications. This study investigated the protective effect of SA on DOX-induced cardiotoxicity in male rats. The ADMETlab 2.0 web server was used to predict the pharmacokinetic properties of SA. Molecular docking studies were conducted using AutoDock Vina. Fifty male rats were divided into 4 groups (*n* = 10); G1 was a negative control; G2 was injected with 4 mg/kg of DOX intraperitoneally (i.p.) once a week for a month; G3 was gavaged by 1/10 of SA LD_50_ (280 mg/kg) daily for a month, and G4 was injected with DOX as in G2 and with SA as in G3. After a month, hematological, biochemical, molecular, and histopathological investigations were assessed. The results showed that SA treatment led to significant amelioration of the DOX-induced cardiotoxicity in rats by restoring hematological, biochemical, inflammatory biomarkers, antioxidant gene expression, and cardiac histopathological alterations. Importantly, the impact of SA treatment against DOX-promoted cardiac deterioration is by targeting the Nrf-2/Keap-1/HO-1/NQO-1 signaling pathway, which in turn induces the antioxidant agents. These findings suggest that SA treatment could potentially mitigate cardiac toxicity during DOX-based chemotherapy.

## Introduction

Chemotherapeutic agents are toxic compounds used to destroy cancerous cells that have significantly improved the likelihood of survival among cancer patients^1^. Doxorubicin (DOX) is a broad-spectrum cytotoxic anthracycline antibiotic that is extensively used in the treatment of several types of malignancies^2^. However, DOX has shown serious side effects, which include neurological disorders, bone marrow aplasia, and especially cardiotoxicity, with a mortality rate exceeding 50% in cases of chronic treatment^3^. Agents that reduce oxidative stress and inflammation have been shown to exert protective effects in preclinical models of DOX-induced cardiotoxicity resulting in the restoration of biochemical, molecular, and histological changes^4,5^. The possible cardioprotective advantages of naturally occurring plant-derived metabolites have garnered significant interest by inhibiting oxidative stress^6^.

Accumulative studies have demonstrated that numerous mechanisms are involved in DOX-induced cardiotoxicity. It has been proposed that DOX-induced cardiac injury is caused by the downregulation of the nuclear factor erythroid-derived (Nrf-2)/heme oxygenase-1 (HO-1) signaling pathway^7^. Naturally occurring phytochemicals have potentially been utilized to induce the Nrf-2 signaling pathway in ameliorating DOX-caused cardiotoxicity^8,9^. A recent study by Cheng et al. (2022) demonstrated that the natural compound glycyrrhetinic acid protects against DOX-induced cardiotoxicity by activating the Nrf-2/HO-1 signaling pathway^10^. Furthermore, it has been reported that cardamonin mitigates DOX-induced cardiotoxicity by upregulating HO-1 and NQO-1^11^. Vascular endothelial growth factor B (VEGF-B) has been shown to have a beneficial effect on endothelial cell survival and maintain heart function, and it can be used as a therapeutic reagent in ischemic injuries. By upregulating VEGF-B, phytochemicals reduce cardiotoxicity in rats^12,13^. Several agents have been explored to mitigate DOX-induced cardiotoxicity, with their respective advantages and disadvantages evaluated. Dexrazoxane is a protective agent approved by the Food and Drug Administration (FDA) for the treatment of DOX cardiomyopathy, but its clinical treatment still has some limitations. Therefore, these limitations highlight the need for the development of new, more effective agents^14,15^.

By enhancing antioxidants, lowering inflammatory cascades, and modulating cell signaling pathways, a variety of phytochemicals have been demonstrated to reduce DOX-induced cardiotoxicity^16^. Among the many different phytochemical classes, shikimic acid (SA) (Trihydroxy cyclohexene carboxylic acid) was first isolated as phenolic from species of *Illicium*, such as *I. anisatum* and *I. verum*^17^. This naturally occurring compound demonstrates a wide range of pharmacological effects, including anti-inflammatory, analgesic, and antioxidant activities, and its action through nuclear factor-kappa B (NF-κB) pathway inhibition and oxidative stress reduction^18^. Furthermore, SA restores defective autophagy and inhibits the MAPK/NF-κB signaling pathway to protect osteoarthritic cartilage^19^. SA also could inhibit the demyelination of nerves through the promotion of oligodendrocyte precursor cell development^20^. The protective effect of SA against cisplatin-mediated kidney injury has been reported^21^. SA has neuroprotective properties and reduces neuro-inflammation in a mouse model of lipopolysaccharide exposure^22^. SA is used as a therapy during blood and liver toxicity and also showed in vivo antihypertensive activity^23,24^. Xing et al. reported the protective effects of SA against acetic acid-induced colitis in rats^25^. Recently, the therapeutic effect of shikimic acid on heat stress-induced myocardial damage has been reported by Gu et al. (2024)^26^. Therefore, it’s critical to assess the beneficial role of SA against DOX-induced cardiotoxicity in rats. More precisely, we investigated how SA affected inflammatory markers and antioxidant defense to better understand their ameliorative effects in DOX-induced cardiotoxicity. Consequently, this study clarified the biochemical processes linked to targeting of the Nrf-2/Keap-1/HO-1/NQO-1 signaling pathway.

## Results

### ADMET properties for DOX and SA

To predict the pharmacokinetic properties of DOX and SA, absorption, distribution, metabolism, excretion, and toxicity (ADMET) were evaluated. The SMILES codes of the compounds were used as input for the ADMETlab 2.0 web server. The ADMET properties suggested that SA exhibits improved solubility (LogS), membrane permeability (Caco-2), and bioavailability (F) when compared to DOX, which may influence its pharmacokinetic profile (Table 1). The toxicity of DOX before and after the reaction with SA was evaluated. The toxicity predictions indicated that SA has a lower probability of inducing cardiotoxicity compared to DOX alone (Table 2). This supports the hypothesis that treatment with SA may mitigate DOX-induced cardiotoxicity.

**Table 1:** ADMET properties for DOX and SA.

Compound	DOX	SA
Log S	− 2.26	− 0.307
Log D	0.473	− 1.129
Log P	1.375	− 1.541
Pgp-inh	0.001	0.001
Pgp-sub	0.998	0.137
HIA	0.829	0.267
F (20%)	0.055	0.009
F (30%)	0.209	0.029
Caco-2	− 6.167	− 5.809
MDCK	6.34E-06	0.00144
BBB	0.015	0.845
PPB	90.86%	19.44%
VDss	1.177	0.334
Fu	10.82%	68.45%
CYP1A2-inh	0.274	0.006
CYP1A2-sub	0.495	0.047
CYP2C9-inh	0.006	0.003
CYP2C9-sub	0.406	0.861
CYP2D6-inh	0.003	0.007
CYP2D6-sub	0.193	0.136
CYP3A4-inh	0.088	0.005
CYP3A4-sub	0.153	0.007
CL	13.025	3.591
T12	0.73	0.917
hERG	0.025	0.009
H-HT	0.261	0.133
DILI	0.974	0.096
Ames	0.829	0.029
ROA	0.041	0.044
FDAMDD	0.323	0.008
Carcinogenicity	0.68	0.019
EC	0.003	0.006
EI	0.012	0.353
Respiratory	0.891	0.035
BCF	0.567	0.074
IGC_50_	3.734	2.177
LC_50_	3.653	2.916
LC_50_ DM	5.55	3.598
NR-AR	0.026	0.022
NR-AR-LBD	0.793	0.014
NR-AhR	0.866	0.007
NR-Aromatase	0.644	0.002
NR-ER	0.217	0.124
NR-ER-LBD	0.588	0.012
NR-PPAR-gamma	0.221	0.023
SR-ARE	0.771	0.013
SR-ATAD5	0.642	0.011
SR-HSE	0.027	0.005
SR-MMP	0.945	0.022
SR-p53	0.979	0.004
MW	543.17	174.05
Vol	516.727	159.75
Dense	1.051	1.09
nHA	12	5
nHD	9	3
TPSA	212.39	94.83
nRot	5	1
nRing	5	1
MaxRing	18	6
nHet	12	5
nRig	28	8
Flex	0.179	0.125
nStereo	5	3
Non-biodegradable	3	1
Toxicophores	3	0
QED	0.147	0.462
Synth	5.015	3.683
Fsp3	0.37	0.714
MCE-18	118.243	22.667

### Molecular docking for DOX and SA

**Fig. 1:**
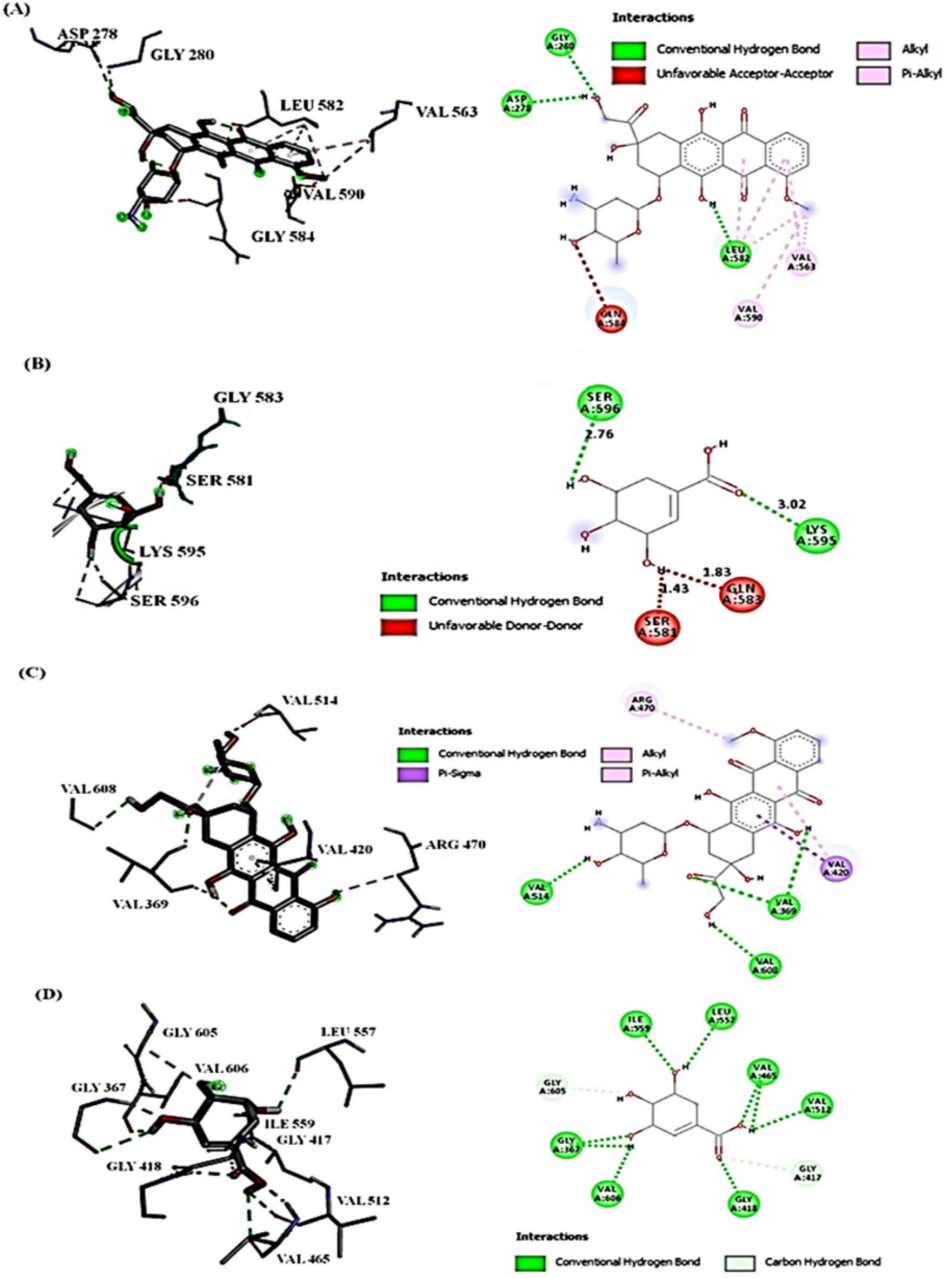
Compounds’ interactions with Nrf-2 and Keap-1 proteins (2D) and (3D). (**A**) DOX interaction with Nrf-2, the conventional H-bond (GLY280, ASP278, LEU582); (**B**) SA interaction with Nrf-2, H-bond (SER596 and LYS595); (**C**) DOX interaction with Keap-1, the conventional H-bond (VAL369 and VAL608), Pi-Sigma (VAL420), and Alkyl (ARG470); (**D**) SA interaction with Keap-1, H-bond (GLY367, VAL465, VAL512, ILE559, and LEU557) and carbon H-bond (GLY605 and GLY417).

Molecular docking results showed the ∆G binding affinities (kcal/mol) for DOX and SA with Nrf-2 and Keap-1 proteins (Table 3). The results indicated that SA exhibited higher binding affinities towards Keap-1 (− 10.6 kcal/mol) compared to DOX. The interaction analyses (Fig. 1) revealed specific interactions between DOX and SA with the target proteins (Nrf-2 and Keap-1). The SA formed multiple hydrogen bonds and hydrophobic interactions with crucial amino acid residues, which potentially lead to more stable and favorable binding (Fig. 1).

**Table 2:** Toxicity of doxorubicin before and after reaction with SA.

Classification	Target	Before		After	
		Prediction	Probability	Prediction	Probability
Organ toxicity	Neurotoxicity	Active	0.60	Inactive	0.55
Organ toxicity	Nephrotoxicity	Active	0.77	Active	0.61
Organ toxicity	Respiratory toxicity	Active	0.88	Active	0.81
Organ toxicity	Cardiotoxicity	Active	0.61	Inactive	0.52
Toxicity end points	Carcinogenicity	Inactive	0.80	Inactive	0.77
Toxicity end points	Immunotoxicity	Active	0.99	Active	0.99
Toxicity end points	Mutagenicity	Active	0.77	Active	0.55
Toxicity end points	Cytotoxicity	Active	0.65	Active	0.50
Toxicity end points	BBB-barrier	Inactive	0.93	Inactive	0.87
Toxicity end points	Clinical toxicity	Active	0.81	Inactive	0.69
Toxicity end points	Nutritional toxicity	Active	0.56	Inactive	0.50
Tox21-Nuclear receptor	Aryl hydrocarbon Receptor (AhR)	Inactive	0.90	Inactive	0.82
Tox21-Nuclear receptor	Aromatase	Inactive	0.73	Inactive	0.60
Tox21-Nuclear receptor	Peroxisome Proliferator Activated Receptor Gamma (PPAR-Gamma)	Inactive	0.97	Inactive	0.94
Tox21-Stress response pathways	Nuclear factor (erythroid-derived 2)-like 2/antioxidant responsive element (Nrf-2/ARE)	Active	0.97	Inactive	0.83
Tox21-Stress response pathways	Heat shock factor response element (HSE)	Inactive	0.97	Inactive	0.96
Tox21-Stress response pathways	Mitochondrial Membrane Potential (MMP)	Inactive	0.59	Inactive	0.70
Tox21-Stress response pathways	Phosphoprotein (Tumor Suppressor) p53	Inactive	0.60	Inactive	0.79
Tox21-Stress response pathways	ATPase family AAA domain-containing protein 5 (ATAD5)	Inactive	0.75	Inactive	0.85
Molecular Initiating Events	Thyroid hormone receptor alpha (THRα)	Inactive	0.90	Inactive	0.90
Molecular Initiating Events	Thyroid hormone receptor beta (THRβ)	Inactive	0.78	Inactive	0.78
Molecular Initiating Events	NADH-quinone oxidoreductase (NADHOX)	Inactive	0.97	Inactive	0.97
Metabolism	Cytochrome CYP1A2	Inactive	0.95	Inactive	0.93
Metabolism	Cytochrome CYP2C19	Inactive	0.84	Inactive	0.73
Metabolism	Cytochrome CYP2C9	Inactive	0.66	Inactive	0.65
Metabolism	Cytochrome CYP2D6	Inactive	0.76	Inactive	0.66
Metabolism	Cytochrome CYP3A4	Inactive	0.92	Inactive	0.83
Metabolism	Cytochrome CYP2E1	Inactive	0.99	Inactive	0.99

**Table 3:** The ∆G binding affinity (kcal/mol) for DOX and SA with Nrf-2 and Keap-1 proteins.

Protein	DOX	SA
Nrf-2	− 7.8	− 5.3
Keap-1	− 8.4	− 10.6

### The oral median lethal dose (LD50) of SA in rats

**Fig. 2:**
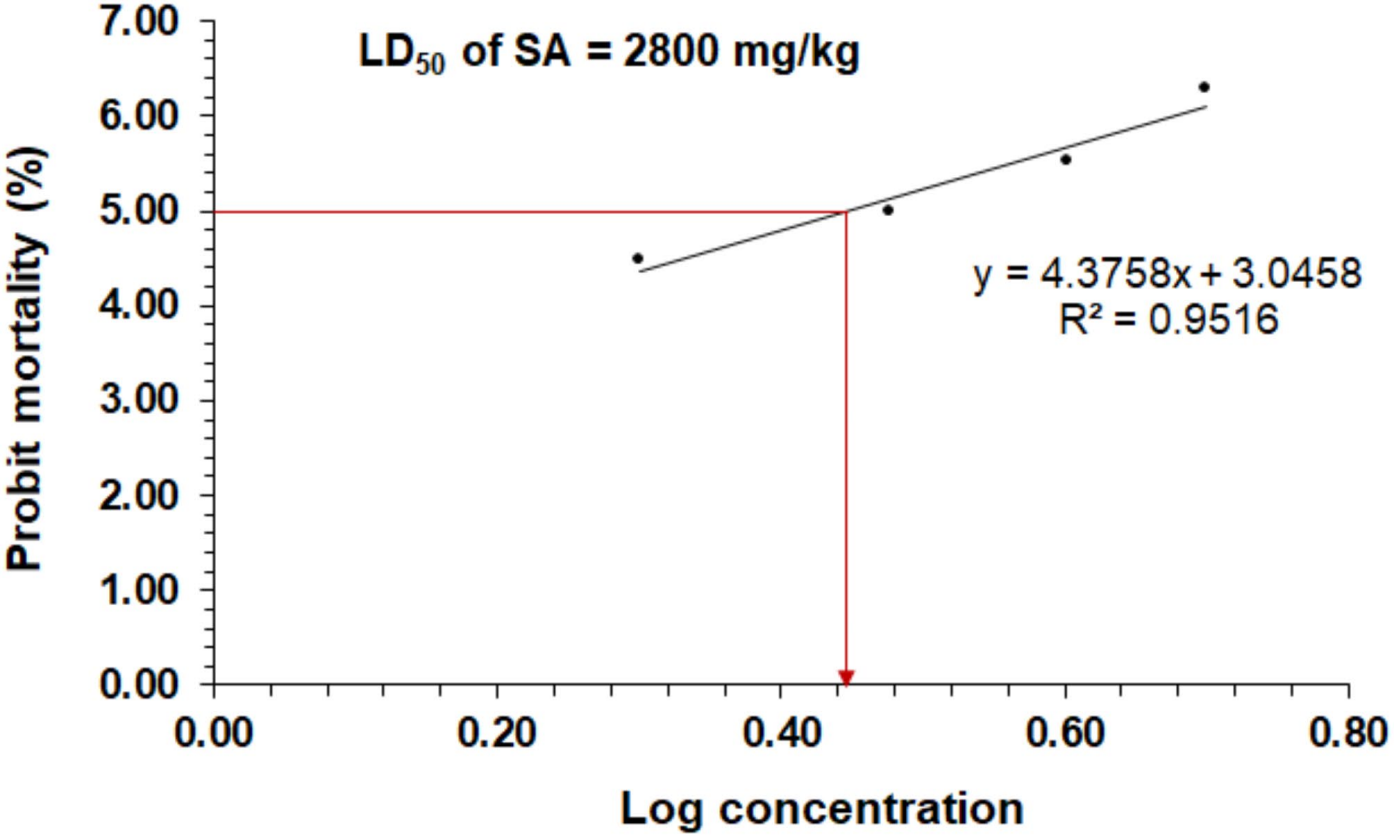
The probit analysis showed the probit mortality (%) in rats following the oral treatment with different doses of shikimic acid (SA), the obtained value of the SA oral median lethal dose (LD_50_) was 2800 mg/kg.

The LD_50_ of SA oral treatment was determined after 24 h using different doses of SA in rats. Different groups were administered different doses ranging from 1000 to 5000 mg/kg vis gavage. The probit analysis showed that the oral LD_50_ of SA was 2800 mg/kg. The animals did not exhibit stereotypical toxic symptoms such as convulsions, ataxia, diarrhea, or increased diuresis except at the 2800 mg/kg dose (see Fig. 2).

### Effect of SA treatment on body weight changes and relative heart weight

**Fig. 3:**
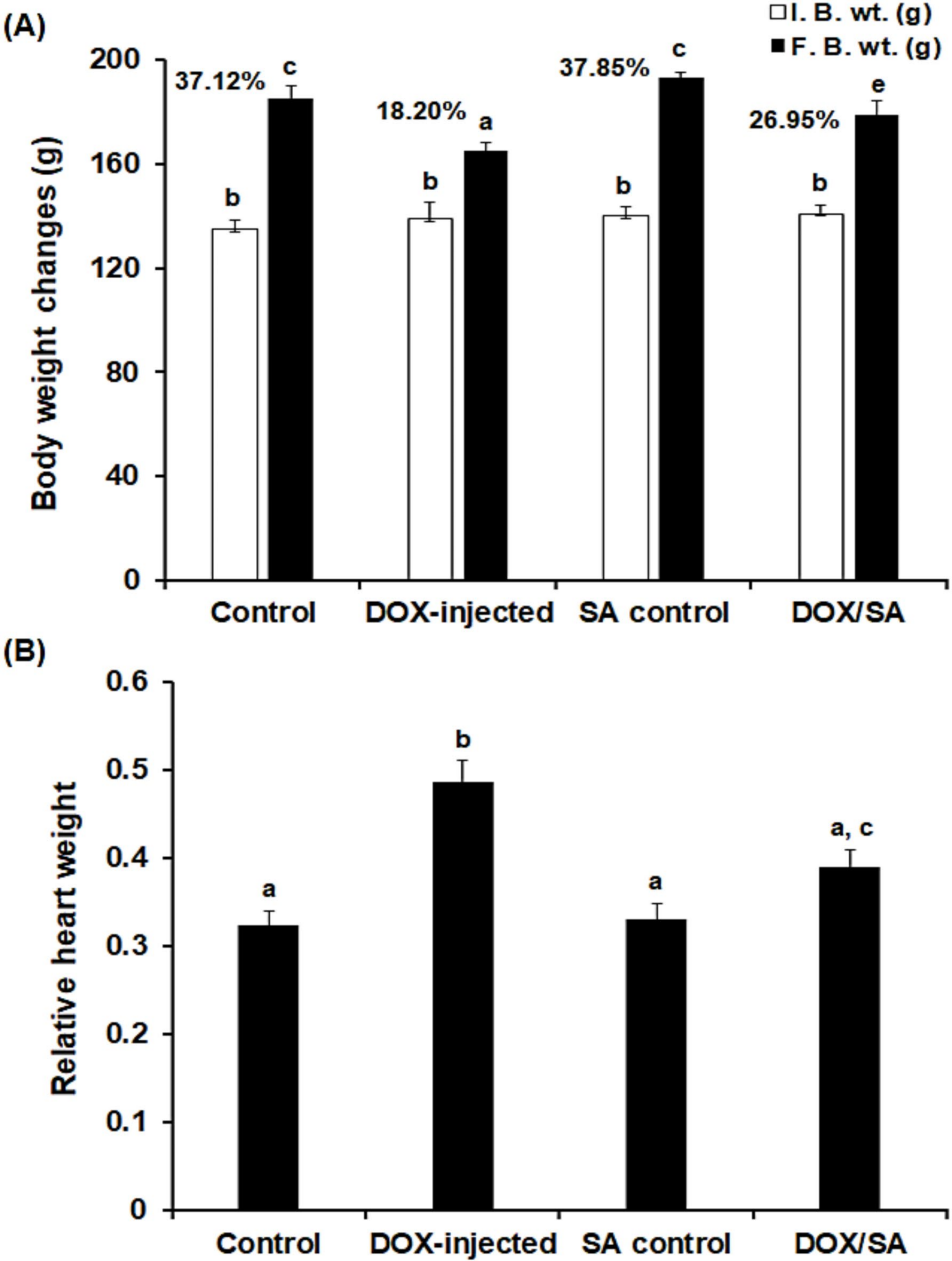
(**A**) The values represent mean ± S.D. (*n* = 10). The body weight changes and (**B**) The relative heart weight in the different groups. *I.B.W* Initial body weight, *F.B.W* Final body weight, *SA* Shikimic acid, *DOX* Doxorubicin. Means that do not share a letter were significantly different (*p* < 0.05).

The results showed a significant decrease (*p* < 0.05) in the percentage of body weight (% BW) change in the group injected with DOX with a decrease up to 18.20% compared to the control group (37.12%) and the SA control group (37.85%). Treating DOX-injected rats with SA led to a significant improvement in the body weight change (%) to 26.95% when compared to the DOX-challenged group alone (Fig. 3A). The ratio of heart weight to body weight in the DOX-injected group significantly increased (*p* < 0.05) compared to the negative control and SA control groups. Treatment of DOX-intoxicated rats with SA resulted in a significant reduction in relative heart weight compared to the DOX-injected group alone (Fig. 3B).

### Effect of SA treatment on cardiac function in isolated hearts

**Fig. 4:**
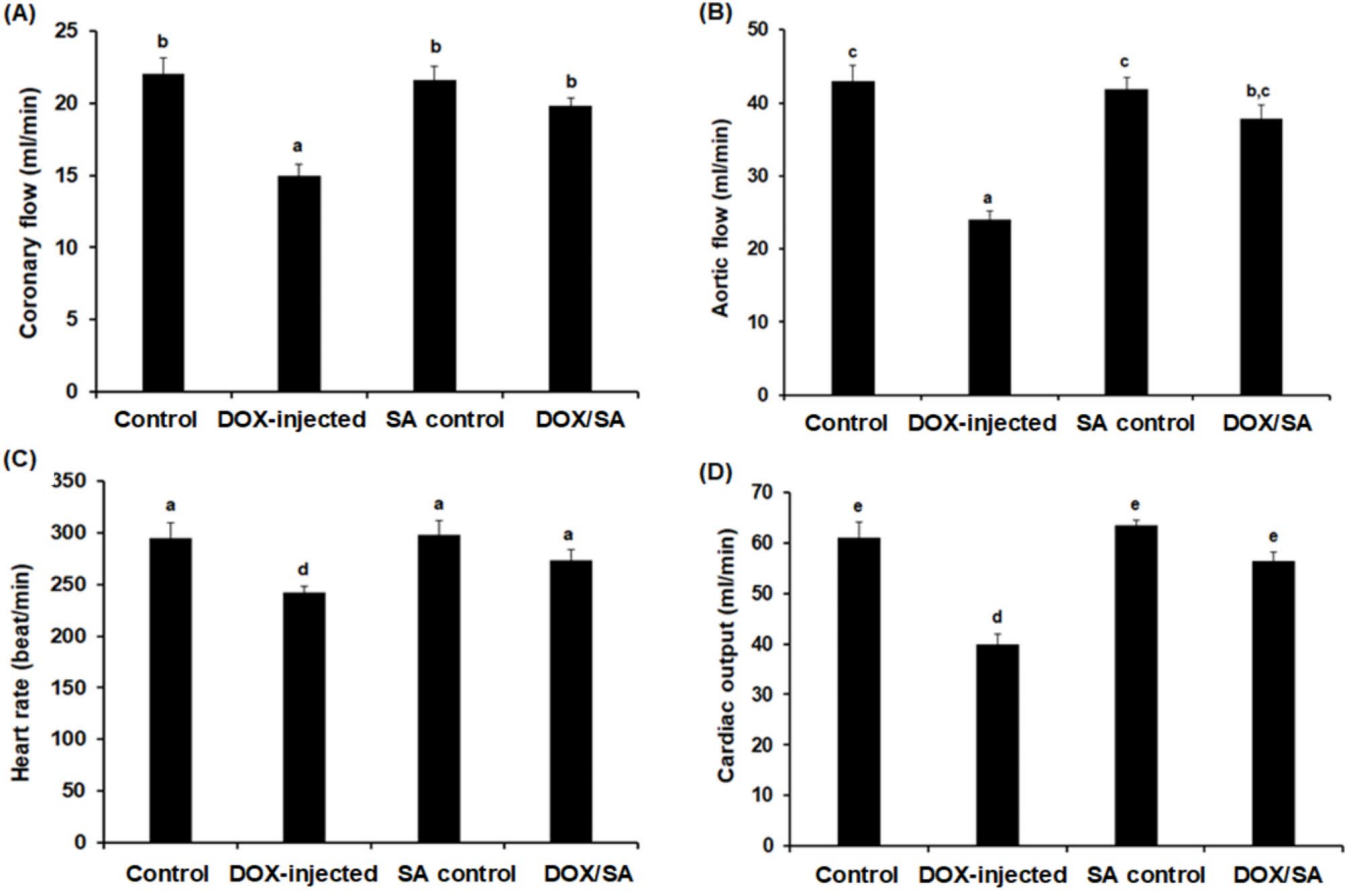
Cardiac functions recorded after 10 min in working mode include (**A**) Coronary flow, (**B**) Aortic flow, (**C**) Heart rate, and (**D**) Cardiac output. Results were provided as average magnitude of each cardiac value within a group of rat ± standard error of mean. (*n* = 10). *SA* Shikimic acid, *DOX* Doxorubicin. Means that do not share a letter were significantly different (*p* < 0.05).

The cardiac function of isolated rat hearts following treatment with DOX and/or SA is depicted in Fig. 4. The coronary flow (CF) and aortic flow (AF) were significantly decreased (*p* < 0.05) in the DOX group compared to the negative control and SA control groups. However, treatment of DOX-intoxicated groups with SA led to a significant increase in CF and AF (*p* < 0.05) when compared to the DOX-group alone. Furthermore, as shown in Fig. 3C, D, there was a significant decrease (*p* < 0.05) in heart rate (HR) and cardiac output (CO) in the DOX-injected group compared to the control groups. The DOX-injected group that was treated with SA revealed significant improvements in HR and CO (*p* < 0.05) when compared to the DOX-injected group alone (Fig. 4).

### Treatment with SA improved hematological alterations that were induced by DOX

The results revealed that the RBC count, Hb levels, platelets, and WBC count were significantly decreased (*p* < 0.001) after injecting 4 mg/kg of DOX once a week for a month compared to the corresponding values of normal control rats. Treatment of DOX-injected rats with SA significantly restored RBC count, Hb levels, platelets, and WBC count close to those in the normal control group (*p* < 0.001) (Tables 4 and 5).

**Table 4:** The hematological parameters in different groups.

Groups	*R*.B.Cs (×10^6^ /µL)	Hb (g/dL)	Hct (%)	Platelets (×10^3^/µL)
Control	7.94 ± 1.15^a^	12.95 ± 1.23 ^a^	37.89 ± 2.25^a^	669 ± 73^a^
DOX-injected	4.06 ± 0.95^b^	8.22 ± 0.91^b^	31.55 ± 2.31^a, b^	569 ± 65^b^
SA control	7.85 ± 0.82^a^	12.68 ± 0.76 ^a^	38.23 ± 3.59^a^	655 ± 76^a^
DOX/SA	6.07 ± 0.74^a, c^	10.95 ± 0.90 ^a, c^	37.16 ± 3.67^a^	594 ± 55^c^

The values represent mean ± S.D. (*n* = 10). *SA* Shikimic acid, *DOX* Doxorubicin. Means that do not share a letter in each column were significantly different (*p* < 0.001).

**Table 5:** The hematological parameters in different groups.

Groups	W.B.Cs (×10^3^/μl)	Lymphocytes (%)	Neutrophils (%)	Monocytes (%)
Control	9.59 ± 1.32^a^	70.25 ± 3.42^a^	25.09 ± 1.56^a^	5.15 ± 0.16 ^a^
DOX-injected	5.12 ± 0.85^b^	84.15 ± 6.18^b^	17.85 ± 3.67^b^	3.21 ± 0.35 ^b^
SA control	8.93 ± 0.83^a^	73.45 ± 3.89^b^	20.44 ± 1.87^b^	4.76 ± 0.14 ^b^
DOX/SA	7.32 ± 1.02^a^	79.32 ± 5.34^b^	19.26 ± 1.87^b^	4.18 ± 0.28 ^b^

The values represent mean ± S.D. (*n* = 10). *SA* Shikimic acid, *DOX* Doxorubicin. Means that do not share a letter in each column were significantly different (*p* < 0.001).

### Treatment with SA significantly modulates biochemical changes induced by DOX

The administration of DOX in rats showed a considerable rise in the levels of CK-MB, cTn-T, AST, LDH, and ANP compared to the control groups. However, when DOX-intoxicated rats were treated with SA, there was a significant reduction in these biochemical parameters close to normal levels compared to the group injected with DOX alone (*p* < 0.001) (Table 6).

**Table 6:** The biochemical parameters in different groups.

Groups	CK-MB (U/L)	cTn-T (ng/ml)	AST (U/L)	LDH (U/L)	ANP (pg/ml)
Control	179.65 ± 7.89^d^	0.84 ± 0.08^a^	67.65 ± 2.34^f^	258.67 ± 15.27^d^	30.47 ± 1.59^a^
DOX-injected	596.56 ± 26.19^c^	2.87 ± 0.15^e^	184.97 ± 3.21^a^	875.93 ± 72.95^e^	58.36 ± 2.53^b^
SA control	186.56 ± 18.42^d^	0.93 ± 0.12^a^	63.74 ± 2.58^f^	242.58 ± 38.89^d^	33.24 ± 1.76^a^
DOX/SA	312.48 ± 23.57^b^	1.45 ± 0.19^c^	94.42 ± 3.07^d^	586.43 ± 66.73^c, f^	46.85 ± 2.78^c^

The values represent mean ± S.D. (*n* = 10). *SA* Shikimic acid, *DOX* Doxorubicin, *CK-MB* Creatine kinase–MB fraction, *cTn-T* Cardiac troponin T, *AST* Aspartate transaminase, *LDH* Lactate dehydrogenase, *ANP* Atrial natriuretic peptide. Means that do not share a letter in each column were significantly different (*p* < 0.001).

### Treatment with SA reduces DOX-induced inflammation by inhibiting inflammatory cytokines

**Fig. 5:**
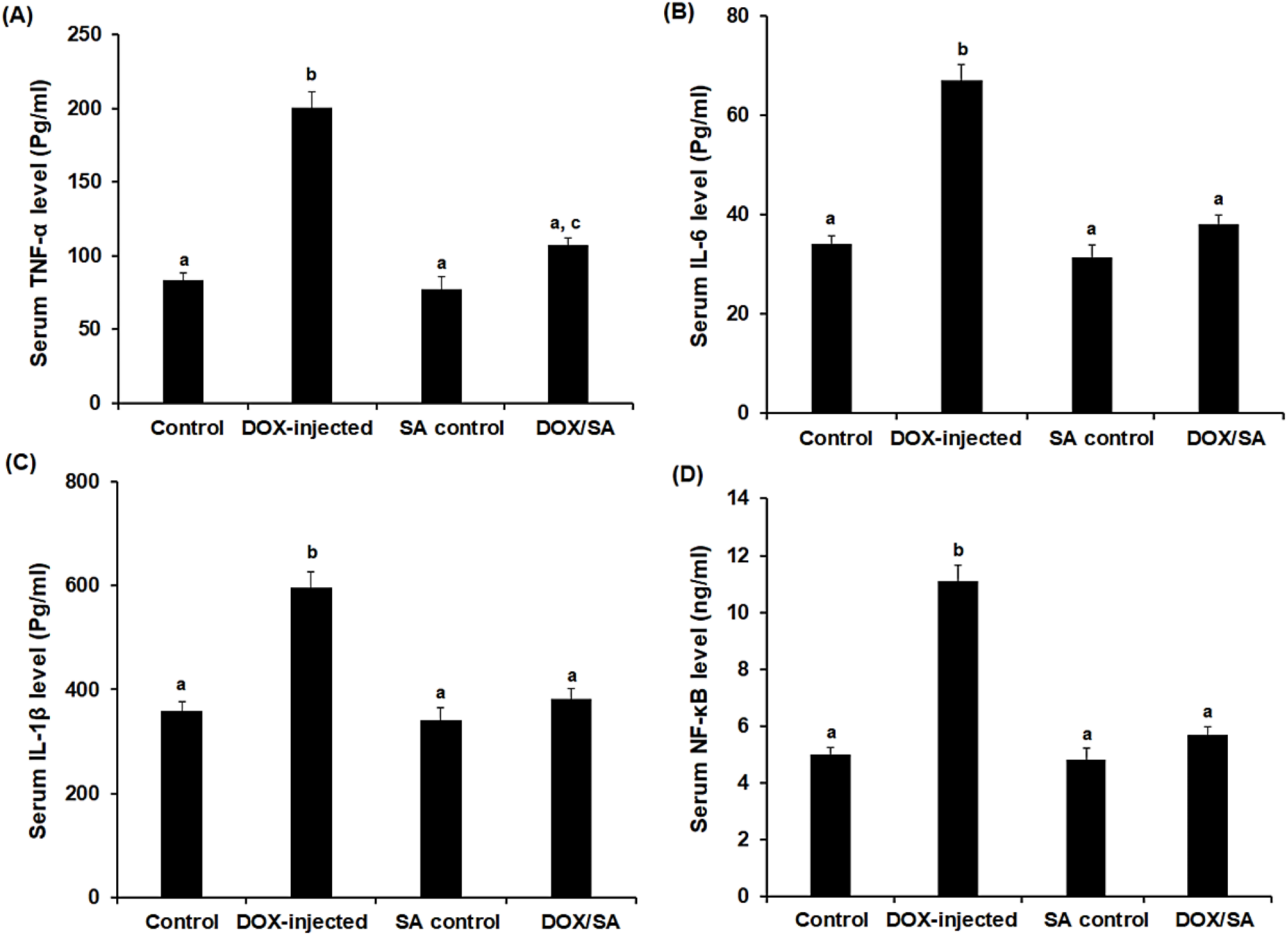
(**A**) Serum tumor necrosis factor alfa (TNF-α), (**B**) Serum interleukin 6 (IL-6), (**C**) Serum interleukin 1 beta (IL-1β), and (**D**) Serum nuclear factor-kappa B (NF-κB) in the different groups. The values represented means ± S.D. (*n* = 10). *SA* Shikimic acid, *DOX* Doxorubicin. Means that do not share a letter were significantly different (p *<* 0.05).

The results showed that serum levels of TNF-α, IL-1β, IL-6, and NF-κB were significantly increased (*p* < 0.05) in the DOX group to 201.45 ± 4.67 Pg/ml, 67.94 ± 2.85 Pg/ml, 597.75 ± 9.98 Pg/ml, and 11.1 ± 0.95 ng/ml, respectively, when compared to the negative control group (84.69 ± 3.87 Pg/ml, 34.75 ± 1.95 Pg/ml, 359.16 ± 5.89 Pg/ml, and 5.6 ± 0.45 ng/ml, respectively). Treatment with SA led to a significant reduction in the levels of the previously mentioned cytokines when compared to the DOX-intoxicated group alone (Fig. 5).

### Administration with SA attenuates DOX-induced cardiac oxidative stress and upregulates VEDF-B

**Fig. 6:**
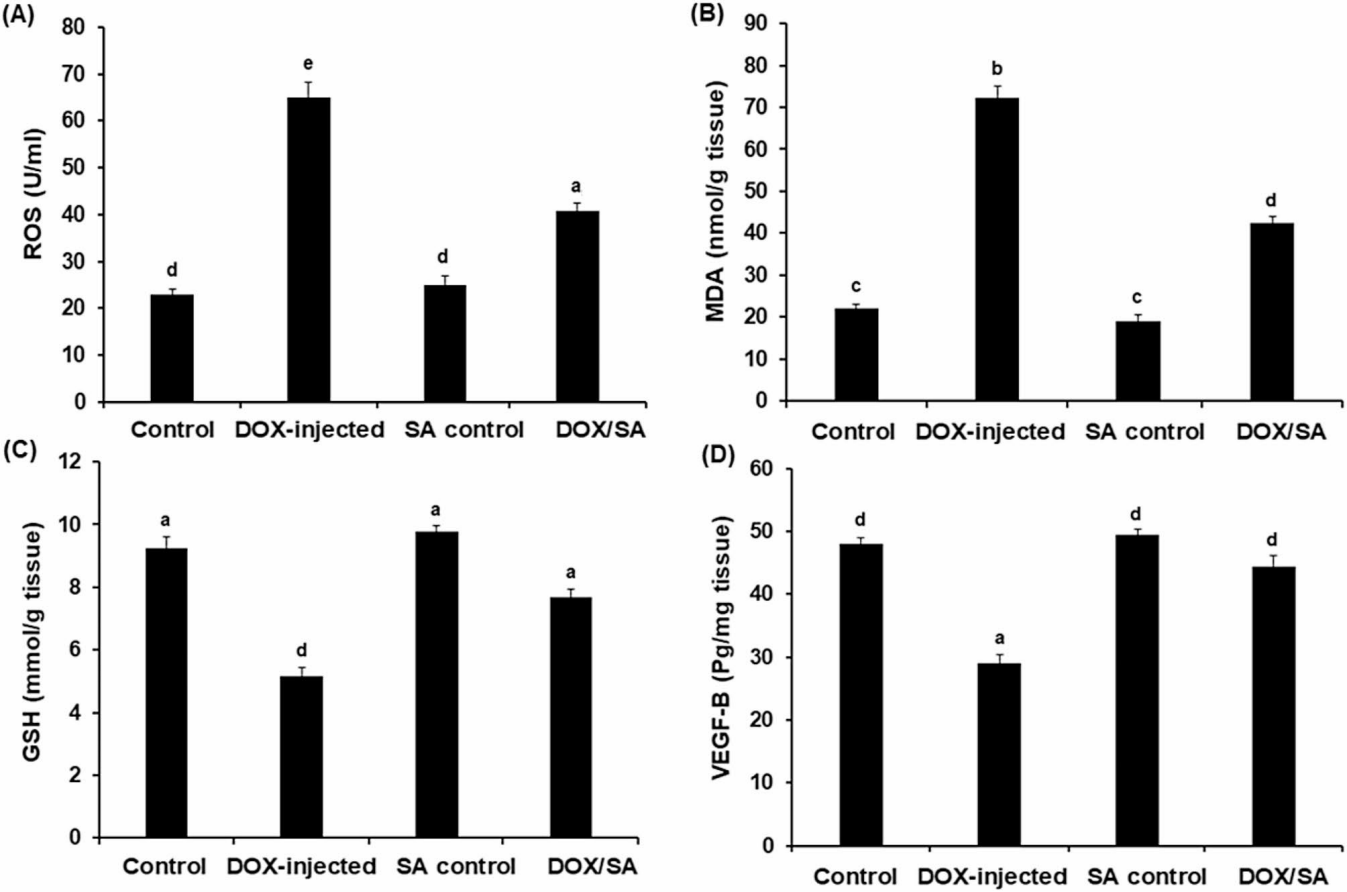
(**A**) Cardiac reactive oxygen species (ROS), (**B**) Malondialdehyde (MDA) levels, (**C**) Reduced glutathione (GSH), and (**D**) Vascular endothelial growth factor B (VEGF-B) in the different groups. The values represented means ± S.D. (*n* = 10). *SA* Shikimic acid, *DOX* Doxorubicin. Means that do not share a letter were significantly different (p *<* 0.05).

Total cardiac reactive oxygen species (ROS) and malondialdehyde (MDA) levels were significantly elevated (*p* < 0.05) in the DOX-intoxicated group reaching 65.32 ± 3.15 U/ml and 73.21 ± 3.52 nmol/g tissue, respectively, compared to the normal control group. Treatment of DOX-injected rats with SA led to a considerable drop in the ROS and MDA levels (Fig. 6). In contrast, rats that were injected with DOX showed a significant decrease (*p* < 0.05) in their cardiac reduced glutathione (GSH) level, which was 5.17 ± 0.46 mmol/g tissue when compared to the normal control group (9.23 ± 1.05 mmol/g tissue). However, the co-treatment with SA led to a significant promotion in cardiac GSH level as compared to the DOX-intoxicated group. Furthermore, the results showed a significant reduction (*p* < 0.05) in the vascular endothelial growth factor B (VEGF-B) levels in the DOX-injected group. Concomitant treatment with DOX/SA restored VEGF-B levels close to their normal values (Fig. 6).

### Treatment with SA reduces DOX-induced cardiac toxicity by modulating Nrf-2/Keap-1/HO-1/NQO-1 pathway in rats

**Fig. 7:**
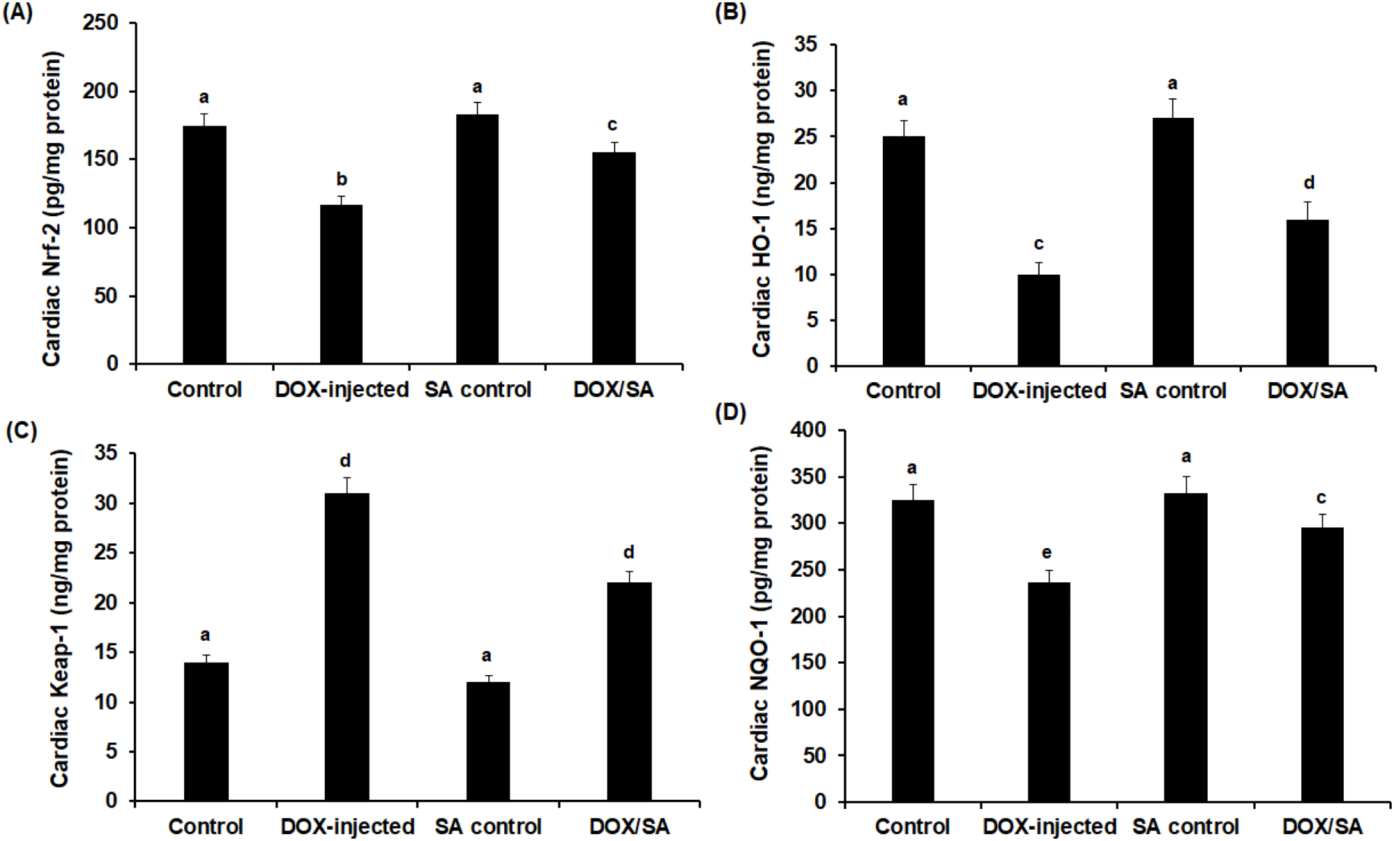
(**A**) Cardiac nuclear erythroid related factor 2 (Nrf-2) (**B**), heme oxygenase-1 (HO-1) (**C**), Kelch-like ECH-associated protein 1 (Keap-1), and (**D**) NAD(P)H: quinone oxidoreductase 1 (NQO-1) in different groups. *SA* Shikimic acid, *DOX* Doxorubicin. The values represented means ± S.D. (*n* = 10). Means that do not share a letter were significantly different (p *<* 0.05).

To elucidate the mechanisms behind the effects of SA treatment on the DOX-induced cardiotoxicity, protein levels and gene expression analysis for the Nrf-2, HO-1, Keap-1, and NQO-1 were evaluated in cardiac tissues after different treatments. The results showed that Nrf-2, HO-1, and NQO-1 protein levels were significantly elevated, while Keap-1 levels were significantly reduced in the cardiac tissues of the DOX-intoxicated rats compared to the control groups. Concomitant treatment with DOX/SA restored Nrf-2, HO-1, Keap-1, and NQO-1 protein levels when compared to the DOX-challenged rats (Fig. 7). The relative mRNA expression of *Nrf-2*, *HO-1*, and *NQO-1* genes was significantly down-regulated (*p* < 0.01); however, the *Keap-1* gene was significantly up-regulated (*p* < 0.01) in the heart tissues of DOX-injected rats in comparison to the control group. Treatment with DOX/SA showed marked restoration of the relative mRNA expression of these genes (Table 7). Furthermore, the relative gene expression levels of the enzymatic antioxidants, including *SOD*, *CAT*, *GPX*, and *GST*, in the cardiac tissues of DOX-intoxicated rats showed significant down-regulations (*p* < 0.001) compared to the control group. Concomitant treatment with DOX/SA led to significant up-regulation of these genes (Table 8).

**Table 7:** Molecular analysis showed the Fold change of nuclear erythroid related factor 2 (*Nrf-2*), heme oxygenase-1 (*HO-1*), NAD(P)H: quinone oxidoreductase 1 (*NQO-1*), and Kelch-like ECH-associated protein 1 (*Keap-1*), genes in different groups.

Groups	Nrf-2	HO-1	NQO-1	Keap-1
Control	1.00 ± 0.03^a^	1.00 ± 0.02^a^	1.00 ± 0.01^a^	1.00 ± 0.01^a^
DOX-injected	0.35 ± 0.04^c^	0.51 ± 0.07^b^	0.42 ± 0.05^b^	3.29 ± 0.17^c^
SA control	1.04 ± 0.07^a^	0.98 ± 0.05^a^	0.97 ± 0.08^a^	1.02 ± 0.06^a^
DOX/SA	0.73 ± 0.08^b^	0.75 ± 0.08^c^	0.79 ± 0.09^a, c^	1.81 ± 0.12^b^

The values represented mean ± S.D. (*n =* 10). *SA* Shikimic acid, *DOX* Doxorubicin. Means that do not share a letter in each column were significantly different (*p* < 0.01).

**Table 8:** Molecular analysis showed the Fold change of superoxide dismutase (*SOD*), catalase (*CAT*), glutathione peroxidase (*GPX*), and glutathione-S-transferase (*GST*), genes in different groups.

Groups	SOD	CAT	GPX	GST
Control	1.00 ± 0.01^a^	1.00 ± 0.01 ^a^	1.00 ± 0.02 ^a^	1.00 ± 0.00 ^a^
DOX-injected	0.27 ± 0.04 ^b^	0.54 ± 0.09 ^b^	0.61 ± 0.08 ^c^	0.59 ± 0.08 ^b^
SA control	1.10 ± 0.16 ^a^	1.25 ± 0.19 ^a^	1.37 ± 0.28 ^a^	1.29 ± 0.35 ^a^
DOX/SA	0.59 ± 0.09 ^c^	0.73 ± 0.08 ^c^	0.85 ± 0.07 ^a, c^	0.76 ± 0.06 ^c^

The values represented mean ± S.D. (*n* = 10). *SA* Shikimic acid, *DOX* Doxorubicin. Means that do not share a letter in each column were significantly different (*p* < 0.001).

### Treatment with SA restores cardiac histopathological alterations that were induced by DOX

**Fig. 8:**
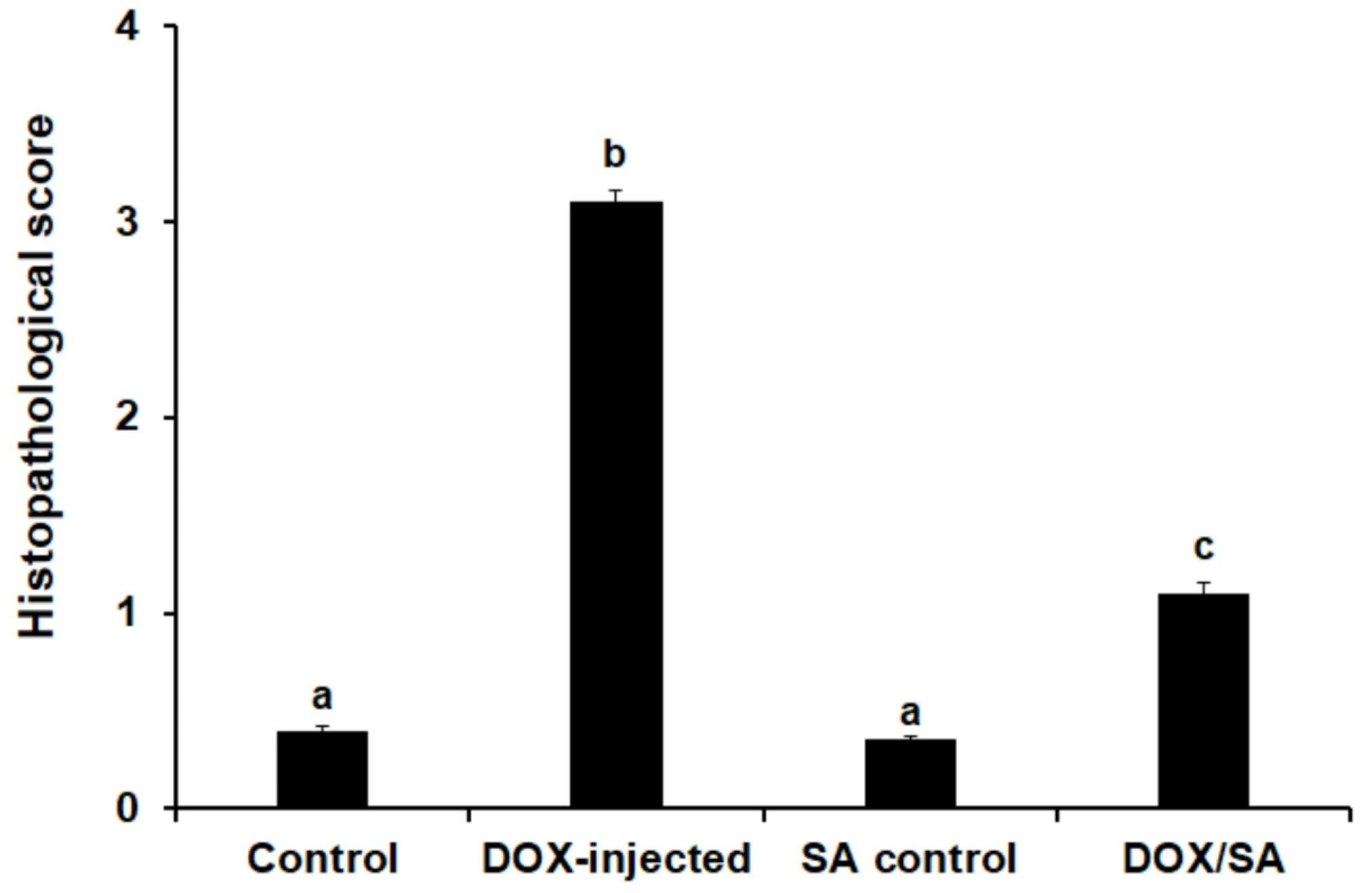
The histopathological score of heart injury in the different groups under the study (*n* = 10). *SA* Shikimic acid, *DOX* Doxorubicin. Means that do not share a letter were significantly different (*p* < 0.01).

**Fig. 9:**
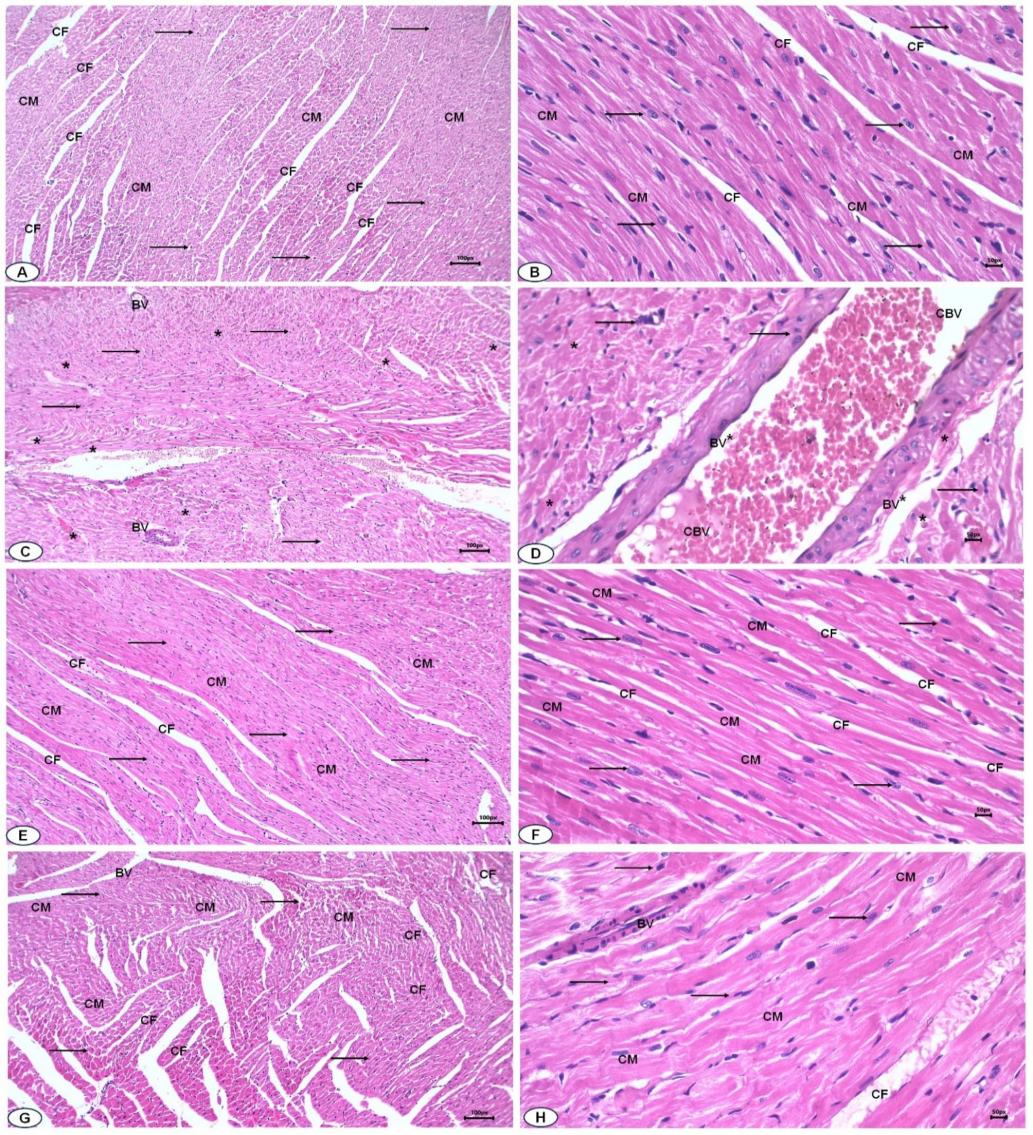
(**A**,** B**) Photomicrograph of heart sections from control group showed arranged cardiac myofibers (CM), normal collagen fibers (CF), and nuclei (arrows). (**C**,** D**) Heart sections from DOX-injected group showed disarrayed cardiac myofibers (*), deformative nuclei (arrows), and congested blood vessels (CBV). (**E**,** F**) Heart sections from SA control group, showed normal-like structures of the cardiac myofibers (CM) and nuclei (arrows). (**G**,** H**) Heart sections from DOX/SA treated group, showed high improvement in the cardiac tissues, organized cardiac myofibers (CM) with centered nuclei (arrows), and less congested blood vessels (BV). (*n* = 10). *SA* Shikimic acid, *DOX* Doxorubicin (H&E ×200, scale bar = 100 μm, H&E ×400, scale bar = 50 μm).

Figure 8 displays the histopathological score of heart damage. Sections of the DOX-challenged group revealed score 3 that exhibited noticeable myofibrillar disruption surpassing 30%, severe damage of contractile components, nuclear deformation, and blood vessels congestion. Treatment with DOX/SA represented a score of 1, indicating less myofibrillar damage with less than 5% of cells exhibited myofibrillar loss, less congestion, and restoration of myofibrillar cross-striation indicating improvements in cardiac histopathological changes that were induced by DOX. Histopathological investigations of H&E-stained heart tissue sections from the negative control group exhibited normal cardiac muscles (CM), single, oval, centrally located nuclei of cardiomyocytes with regularly arranged cardiac architectures; and dense cytoplasm with normal collagen fibers (CF) (Fig. 9A, B). Heart tissue sections from the DOX-injected group exhibited severe histopathological alterations in the cardiac tissues compared to the control groups; certain areas show loss of cross-striation and disruption of the cardiac cellular architecture, as well as deformation of some nuclei. Moreover, the cardiac myofibers were found to be in a disorganized pattern. The blood vessels were highly damaged and congested compared to the normal control group (Fig. 9C, D). Heart sections from the SA control group showed a normal-like cardiac tissue architecture, aligned collagen fibers (CF), and nuclei with normal blood vessels (BV) (Fig. 9E, F). Heart sections from the DOX/SA-treated group showed significant improvement in the cardiac tissues; with organized cardiac myofibers (CM), collagen fibers (CF), oval, centered nuclei, and less congested blood vessels (BV) (Fig. 9G, H).

## Discussion

Doxorubicin (DOX) has been associated with fatal cardiotoxic adverse effects resulting in left ventricle dysfunction and the progression of congestive heart failure, leading to a high mortality rate in chronic use of DOX^27^. Enhanced oxidative stress is one of the main contributions to the multifactorial underlying mechanism of DOX toxicities^28,29^. Therefore, it’s necessary to find new, safe chemo-protective agents that could reduce DOX’s harmful effects. Due to their high antioxidant activity and general toleration, phytochemicals could counteract the cardiac damaging effects of DOX^30^. SA has been shown to reverse the effects of oxidative stress due to its potential antioxidant activities and protect vital tissues against chemotherapeutic toxicity^21^. The biological functions and molecular mechanisms of SA have been reported; however, few studies have reported the beneficial effects of SA on cardiac injuries^31^. A previous study investigated the therapeutic potential of SA on myocardial damage, indicating that SA significantly mitigated the inflammatory response and modulated the PI3K/AKT signaling pathway^26^. The neuroprotective effect of SA has been investigated against hydrogen peroxide-induced oxidative stress on human neuronal-like cells (SH-SY5Y)^32^. The current study provides for the first-time new evidence of the cardioprotective efficacy of SA treatment against DOX-induced cardiotoxicity, which may be mediated via inhibiting oxidative stress, inflammation, and Nrf-2/Keap-1 signaling pathway modulation.

The results indicated that SA exhibits higher binding affinities towards both Nrf-2 and Keap-1, suggesting an enhanced ability to modulate the Nrf-2/Keap-1 signaling pathway. This pathway plays a crucial role in regulating cellular antioxidant defenses and protecting against oxidative stress-induced cardiotoxicity. The interaction analysis demonstrated that the SA forms multiple hydrogen bonds with crucial amino acid residues in Keap-1. These interactions could contribute to the stable binding and inhibition of Keap-1 by the product. Under normal conditions, Keap-1 binds to Nrf-2 and promotes its ubiquitination and proteasomal degradation. However, when Keap-1 is inhibited, Nrf-2 accumulates in the cytoplasm and translocated to the nucleus. There, it activates the transcription of various cytoprotective genes involved in the antioxidant defense system (HO-1, NQO-1). The strong binding of the product to Keap-1, as indicated by the molecular docking results and interaction analysis, suggests that the SA may disrupt the Keap1-Nrf-2 interaction. This inhibition of Keap-1 by the product can lead to the accumulation and nuclear translocation of Nrf-2, resulting in the activation of the Nrf-2-mediated antioxidant response pathway. By activating the Nrf-2 signaling pathway through the inhibition of Keap-1, the product could enhance cellular antioxidant defenses and protect against oxidative stress-induced cardiotoxicity caused by DOX. The upregulation of cytoprotective enzymes, including HO-1 and NQO-1, could contribute to the mitigation of DOX-induced cardiotoxicity^33,34^.

The results showed a significant decrease in the body weight change percentage and notable increase in heart weight of the DOX-challenged group. This could be due to the toxic effects of DOX leading to reduced appetite, inhibition of protein production, and disruption of basal metabolism^35^. Treatment with SA reinstated the body weight and heart weight changes in rats injected with DOX suggesting the protective effects of SA against DOX toxicity. This finding aligned with previous studies that reported the positive impacts of natural products on improving body weight change in experimental animals^35,36^. The DOX-intoxicated groups that were treated with SA showed significant improvements in cardiac functions in the hearts isolated from the animals, including heart rate and cardiac output 24 h after the last dose of SA and/or DOX. These findings were in accordance with a previous study that demonstrated the cardioprotective effect of natural constituents on DOX-induced cardiotoxicity^37^.

The results obtained from the present investigation showed that treatment with SA restored the hematological parameter alterations induced by DOX injection. The reduction in hematological parameters following DOX treatments may result from hematopoietic system injury or increased cell membrane permeability, leading to erythrocyte hemolysis and osmotic edema. Additionally, the decreased hemoglobin affinity for oxygen induces hypoxia^38^. A previous study reported the positive impacts of polyphenols treatment against hematological parameter disturbance induced by DOX in experimental rats^39^. Furthermore, in the present study, cardiac injury biomarkers, including CK-MB, cTn-T, AST, LDH, and ANP, were released from the cardiac tissue into the bloodstream following DOX injection when myocardial cells became damaged due to increase in cell membrane permeability. Treatment with SA resulted in a significant reduction in the serum levels of these markers indicating the cardioprotective potential of SA treatment^40^. In this study, DOX-injected rats showed a significant increase in serum proinflammatory cytokines, including TNF-α, IL-6, IL-1β, and NF-κB. Consistently, previous studies have shown that DOX treatment increases pro-inflammatory cytokines including TNF-α, IL-6, and IL-1β, in cardiac tissue through activation of NF-κB, triggering the progression of adverse cardiac events^41,42^. Indeed, treatment with SA led to a significant reduction in the previously mentioned cytokines in comparison to single treatment, which confirms the anti-inflammatory effects of SA treatment against DOX-induced inflammation. In accordance with a previous study by Nagoor Meeran et al. (2023), which reported that α-Bisabolol attenuated DOX-induced acute cardiotoxicity in rats by inhibiting inflammatory cascades^43^. Another study reported that SA could inhibit cellular pro-inflammatory cytokine production and alleviate mechanical hyperalgesia in mice^44^.

Treatment with DOX induces oxidative stress due to an imbalance between the antioxidant defense system and the activation of ROS, which is one of the fundamental causes of DOX-dependent cardiotoxicity, leading to severe cellular injury^45^. The results obtained in the current work indicated significant antioxidant activity of SA treatment, as evidenced by the amelioration of pathological alterations induced by DOX injection to the oxidative stress markers, including ROS, MDA, and GSH, in rats’ cardiac tissues. This could be due to decreasing the susceptibility of cardiomyocytes to ROS, enhancing GSH biosynthesis, and inhibiting MDA formation. These findings suggested that SA treatment could protect against DOX-induced oxidative stress in the heart tissues of rats by deactivating ROS, consistency with previous studies that reported the significance of natural products treatment against DOX-induced cardiac tissue damage in experimental animals^30,46,47^. Vascular endothelial growth factor (VEGF-B) could protect cardiac tissues by developing collateral coronary arteries and inducing new blood vessel growth^48^. In the present study, the treatment with SA could upregulate the VEGF-B in the heart tissues of DOX-intoxicated rats, which caused new blood vessels to develop and nourish the injured cardiac muscles, reducing the cardiotoxic effects of DOX. A previous study reported that citronellol natural compounds reduced cardiac injury caused by DOX injection and upregulated VEGF expression in rats^48,49^.

The Nrf-2 is an essential modulator of numerous physiological and pathological processes, primarily involved in controlling the redox status of cells^50^. The efficient antioxidant networks that protect against oxidative stress include both inducible detoxifying enzymes, such as HO-1 and NQO-1, through the activation of Nrf-2, and primary enzymes, such as SOD, CAT, and GPx. Interestingly, under physiological conditions, Nrf-2 is sequestered by binding to Keap-1, which inhibits the translocation of Nrf-2 into the nucleus. Keap-1 undergoes a conformational shift in response to various inducers, which releases Nrf-2, causing it to bind to the antioxidant-related elements in the promoter regions of genes associated with cyto-protection and antioxidants^51,52^. Numerous cellular processes are regulated by redox homeostasis in both healthy and diseased settings. Particularly, in the heart tissues, NRF-2 has been identified as a crucial modulator of cellular defense against a range of injuries. The HO-1 is an inducible form; its expression is increased by oxidative stress^53^. Induced HO-1 is thought to act as an antioxidant defense mechanism through degrading cellular heme and increasing antioxidants as well as playing a role in suppressing various inflammatory responses^54^. Interestingly, the current study showed that concomitant treatment with DOX/SA led to significant upregulation of cardiac *Nrf-2*,* HO-1*, and *NQO-1* genes and protein levels in comparison to DOX-intoxicated rats, however, *Keap-1* was down-regulated. A previous study demonstrated that Nrf-2 reduced cardiovascular associated risk factors^55^. These findings were in accordance with previous studies reported the significant role of natural products in modulating the Nrf-2/HO-1 pathway and their downstream antioxidant related signaling in heart tissues of experimental animals indicating that targeting Nrf-2 could help to relieve DOX-induced cardiotoxicity^9,43,56–58^.

Heart tissue sections from the DOX-injected group showed severe histopathological alterations in the cardiac tissues, including disruption of cardiac muscle structure, deformation in nuclei shapes indicating dead cells, myofiber disarray, and extensive damage to blood vessels. These changes may be due to the degeneration of structural proteins suggesting substantial cardiac injury and dysfunction induced by DOX toxicity. However, heart sections from the DOX/SA-treated group showed significant improvements in cardiac myofibers with visible nuclei and less congested blood vessels, which could suggest better vascular health or reduced inflammation. These improvements indicate the positive effects of SA treatment on heart tissue, potentially protecting against damage caused by DOX. These improvements in cardiac histology support the cardioprotective effect of SA that was confirmed by the reduction of the oxidative stress and infiltration of inflammatory cells, and cytokines. These improvements strongly correlate with the observed molecular and functional outcomes, suggesting an integrated cardioprotective mechanism of SA. The activation of Nrf-2 leads to its translocation into the nucleus by Keap-1, which controls oxidative and electrophilic stress by the cellular response and manages the activity of gene detoxification, expressing several antioxidants including GSH enhancing cardiac tissues regeneration. Furthermore, the structural restoration in cardiac tissues aligns with Nrf-2/Keap-1 pathway activation, leading to increased expression of HO-1 and NQO1 antioxidant enzymes, which reduces oxidative stress, inhibits lipid peroxidation that evidence by reduction of the cardiac MDA levels, and mitigates cardiac histological damage^33,59^. These findings suggested the mechanistic basis of SA’s cardioprotective effects, which were consistent with previous studies that demonstrated the effective role of the treatment with natural constituents against DOX-induced cardiac tissue damage in experimental animals^60–62^. The enhanced antioxidant response likely contributed to the preservation of myocardial architecture by reducing oxidative damage to cardiomyocytes and inhibiting apoptotic signaling pathways. The observed histopathological improvements, such as well-preserved myofibrillar structure and reduced interstitial fibrosis, further support this mechanism. By mitigating oxidative stress-induced cellular damage, SA helped maintain tissue integrity, ultimately preventing the progression of structural deterioration in the heart. Functionally, the restoration of cardiac histological architecture was accompanied by significant improvements in cardiac output and coronary flow. The structural preservation of myocardial tissue is likely translated into enhanced myocardial contractility and vascular efficiency. Moreover, reduced fibrosis and preserved microvascular integrity may have contributed to better coronary perfusion, ensuring optimal oxygen and nutrient delivery to cardiac tissues. These functional benefits align with the upregulation of Nrf-2 and antioxidant defense mechanisms, suggesting that SA’s protective effects extend beyond molecular signaling to tangible physiological improvements.

## Conclusion

Collectively, SA, a natural herbal compound, has demonstrated a range of pharmacological properties in preclinical studies. These include anti-inflammatory, antioxidant, and immunomodulatory effects, making it a promising agent for therapeutic applications. In this study, the daily administration of SA at sub-lethal doses (1/10 of the LD_50_) in rats for a month significantly ameliorated the cardiotoxicity induced by DOX in rats. This investigation provided a comprehensive understanding of the mechanisms underlying SA’s cardioprotective effects. The interplay between molecular signaling (Nrf-2 activation), structural preservation (histopathology), and functional enhancement (cardiac output and coronary flow) underscores the therapeutic potential of SA in mitigating doxorubicin-induced cardiotoxicity. These findings could provide a new strategy for efficient cancer chemotherapy. However, human data on the direct use of SA is limited; therefore, further preclinical and clinical studies should investigate the effects of SA on different cardiotoxicity models, studying its pharmacokinetics for optimizing its usage in clinical settings to maximize therapeutic efficacy and minimize toxicity. Therefore, in vivo studies will be performed in future studies to investigate the pharmacokinetics of SA for usage in cardiotoxicity therapy against DOX.

## Materials and methods

### Chemicals

Doxorubicin hydrochloride (catalog no. D5220, 98–102% HPLC) was purchased from Sigma-Aldrich (Oakville, ON L6H 6J8), Canada. Shikimic acid (SA) (catalog no. S5375, ≥ 99%) was purchased from Sigma-Aldrich (St. Louis, MO), USA. SA was dissolved in phosphate-buffered saline (PBS) and stored at 4 °C in the dark.

### In silico ADMET prediction

The structures of doxorubicin and shikimic acid were retrieved from PubChem (CIDs: 31703, 442428, and 8742, respectively) in SDF format. The reaction between these compounds was predicted using the Forward Reaction Prediction tool on the RXN IBM platform (https://rxn.res.ibm.com/). The resulting product was obtained as a SMILES code. The SMILES codes of the compounds (doxorubicin and shikimic acid) were used as input for the ADMETlab 2.0 web server to predict their pharmacokinetic properties, including absorption, distribution, metabolism, excretion, and toxicity (ADMET)^63^. The toxicity of the predicted reaction product and doxorubicin was further evaluated using the ProTox-III server. The SMILES codes of these compounds were used as input for the toxicity prediction^64^.

### Molecular docking

AutoDock Vina was used to perform molecular docking studies that predict the binding modes and affinities of SA with Nrf-2 and Keap-1. The grid boxes for docking were centered on the predicted binding sites. The structures of the compounds (doxorubicin and shikimic acid) were energy-minimized using Avogadro 1.2.0 software with the MMFF94 force field^65^. The sequences of the Nrf-2 (UniProt ID: O54968) and Keap-1 (UniProt ID: P57790) proteins were retrieved from the UniProt database. The binding sites for these proteins were predicted based on literature information and validated using the DeepSite web server on the PlayMolecule platform (https://www.playmolecule.org/). The proteins were prepared for docking using AutoDock Tools 1.5.7, which involved removing water molecules, adding polar hydrogens, and assigning Gasteiger charges^66^. The exhaustiveness parameter was set to 8, and the default scoring function was used for the docking calculations. The docked complexes were analyzed using BIOVIA Discovery Studio Visualizer 2020 (Dassault Systèmes BIOVIA, 2020). The binding affinities (ΔG values) and intermolecular interactions, including hydrogen bonds and hydrophobic interactions, were analyzed and reported.

### Rats and experimental design

Fifty male Sprague-Dawley rats (130–150 g, 5–6 weeks of age) were purchased from Helwan University, Egypt. The study was conducted in accordance with the guidelines of the Declaration of Helsinki and ARRIVE (2.0) guidelines that followed the experimental design, bias minimization, sample size and statistical analyses. Rats were randomly divided into 4 groups (5/cage). Target values for temperature and relative humidity were approximately 22 ± 1 °C and 55 ± 5%, respectively, with a light-dark (day/night) cycle established. The rats had access to tap water and standard pelleted animal food ad libitum. The experimental protocol was approved by Tanta University’s Faculty of Science’s Animal Care Committee with approval number (IACUC-SCI-TU-0400), Egypt. The rats were divided into 4 groups (*n* = 10); G1 was a negative control group that orally injected with PBS daily; G2 was injected with 4 mg/kg of DOX i.p. once a week for a month^67^; G3 was gavaged daily with 280 mg/kg of SA (1/10 of SA LD_50_); G4 was injected with DOX as in G2 and with SA as in G3. After a month, all groups had been anesthetized by isoflurane. Blood samples were drawn from the left jugular vein, and then the heart was excised after a thoracotomy under terminal anesthesia. The Langendorff “non-working” method of perfusion was applied while the aorta was cannulated. The perfusion medium was Krebs-Henseleit bicarbonate buffer that had been oxygenated. The apparatus was put into operating mode, and the pulmonary vein was cannulated, as previously described by Tosaki and Hellegouarch^68^. After 10 min of aerobic perfusion, the basic cardiac function was registered. Thus, the heart rate (HR) was registered using a computer acquisition system (AD Instruments, PowerLab, Castle Hill, Australia). Coronary flow and aortic flow (AF) were measured by a calibrated flowmeter. Cardiac output (CO) was calculated as the sum of AF and CF. The percentage of body weight changes and relative heart weight were calculated. Separated serum samples were used for biochemical tests. The excised hearts were immediately rinsed into cold PBS to remove blood and cut into pieces, then homogenized in PBS for biochemical and molecular analyses. Also, heart tissues were sectioned for histopathological studies in buffered formalin.

### Hematological and biochemical analysis

Hematological parameters, including red blood cells (RBC), hemoglobin (Hb) content, white blood cells (WBC), differential count, and platelets, were estimated using standard automated procedures. Biochemically, serum creatine-kinase MB (CK-MB) and lactate dehydrogenase (LDH) enzymes were determined using kits (Thermo Scientific, Waltham, MA, USA) following the manufacturer’s instructions, CK-MB (catalog no. AKC0325) and LDH (catalog no. C20300). Serum aspartate aminotransferase (AST) (catalog no. AS106145) was measured by Bio-diagnostic kits, Egypt. A rat ELISA kits from MyBioSource, Inc., San Diego, CA, USA, was used to measure serum levels of cardiac troponin T (cTn-T) (catalog no. MBS039759) and atrial natriuretic peptide (ANP) (catalog no. MBS2513078) following the manufacturer’s guidelines. Furthermore, serum TNF-α (catalog no.: E-UNEL-R0057), IL-6 (catalog no.: E-EL-R0015), and IL-1β (catalog no.: E-EL-R0012) were determined by using their specific rat’s ELISA kits from Elabscience (Houston, Texas, USA). ROS production in rats’ heart tissues was determined using an ELISA kit (MBS164653; MyBioSource Co., San Diego, CA, USA) following the manufacturer’s instructions. Malondialdehyde (MDA), and reduced glutathione (GSH) levels (catalog no. MD2529 and catalog no. GR2511, respectively) were measured in the heart tissue homogenate using their Kits (Biodiagnostic, Egypt)^69,70^. Moreover, serum NF-κB (catalog no. MBS287521), and cardiac VEGF-B (catalog no. MBS161946) were evaluated using rat’s ELISA kits from MyBioSource (Inc., San Diego, CA, USA). Cardiac protein levels of Nrf-2 (catalog no. MBS752046), HO-1 (catalog no. MBS764989), Keap-1 (catalog no. MBS7218529), and NQO-1 (catalog no. MBS9337618) were determined using their rat’s ELISA kits from MyBioSource (Inc., San Diego, CA, USA).

### Molecular analysis

Total RNA was extracted from ventricular samples of cardiac tissues using TRIzol reagent (Thermo Fisher Scientific) according to the manufacturer’s instructions. SYBR Green was used to assess the mRNA’s expression of *Nrf-2*,* HO-1*,* NQO-1*,* Keap-1*, *SOD*, *CAT*, *GPX*, and *GST* genes. Normalization against the GAPDH reference gene was made, and the primers used are shown in Table 9. The mRNA levels were detected on a LightCycler 480 System (Roche, Indianapolis, IN) under the following cycling conditions: 1 cycle at 95 °C for 5 min followed by 45 cycles at 95 °C for 10 s, 60 °C for 30 s, and 72 °C for 10 s. Fold changes were used to compare the differences between the groups. The relative gene expression was calculated by the method of Livak and Schmittgen (2001)^71^.

**Table 9:** Forward and reverse oligonucleotide primers sequences used for RT-qPCR.

Gene	Accession number	Forward sequence (5′–3′)	Reverse sequence (5′-3′)
*Nrf-2*	NM_001399173	TTGTAGATGACCATGAGTCGC	CAGGGGTGGTGAAGACTGAG
*HO-1*	NM_012580	TCAACATTGAGCTGTTTGAG	AAATTCCCACTGCCACGGT
*NQO-1*	NM_017000	CGCAGACCTTGTGATATTCCAG	TGTTGCGCTCAATCTCCTCCT
*Keap-1*	NM_057152	CATCGGCATCGCCAACTTC	GCTGGCAGTGTGACAGGTTGA
*SOD*	NM_017050	ACCGAGGAGAAGTACCACGA	TAGGGCTCAGGTTTTGTCCAG
*CAT*	NM_012520	CCTCAGAAACCCGATGTCCTG	GTCAAAGTGTGCCATCTCGTCG
*GPX*	NM_030826	TGAGAAGTGCGAGGTGAATG	CGGGGACCAAATGATGTACT
*GST*	NM_017014	GCTGGAGTGGAGTTTGAAGAA	GTCCTGACCACGTCAACATAG
*GAPDH*	NM_031144	TTCCAGGAGCGAGATCCCGCTAAC	CATGAGCCCTTCCACGATGCCAAAG

### Histopathological investigations

Heart sections from the left ventricle that had been fixed with formalin were treated with various grades of alcohol and xylene before being embedded in paraffin blocks. Sections stained with hematoxylin and eosin (H&E) were examined under a light microscope (Olympus CX31, Tokyo, Japan) to determine cellular damage^72^. Four sections were obtained at standardized thicknesses (5 μm). From each section, 20 photos were captured under consistent magnification, (H&E ×200, scale bar = 100 μm, H&E ×400, scale bar = 50 μm) (10 photos for each magnification) ensuring representative sampling of the tissue architecture. The degree of cardiac tissue deterioration in different sections was scored on a scale of 0–3. When myofibrillary degeneration was absent, a score of 0 was given. A score of 1 showed less than 5% of cells exhibited myofibrillar loss. For 15–30% of cells exhibiting notable myofibrillar loss and/or cytoplasmic degeneration, a score of 2 was assigned. Most cardiac myocytes showed a myofibrillar breakdown, nuclear deformation, blood vessels congestion and a discernible loss of contractile components, earning a score of 3 for diffuse damage surpassing 30%.

### Statistical analysis

The one-way ANOVA results were analyzed using Graph Pad Prism software (San Diego, CA) (https://www.graphpad.com/). Tukey was then employed for multiple comparisons to ascertain the significance of the differences between the treatment groups and the control; *p* < 0.05 was considered acceptable significance.

## Data Availability

The datasets used and analyzed during the current study are available from the corresponding author on reasonable request.
